# Detection of Mild Cognitive Impairment Through Natural Language and Touchscreen Typing Processing

**DOI:** 10.3389/fdgth.2020.567158

**Published:** 2020-10-08

**Authors:** Anastasia Ntracha, Dimitrios Iakovakis, Stelios Hadjidimitriou, Vasileios S. Charisis, Magda Tsolaki, Leontios J. Hadjileontiadis

**Affiliations:** ^1^Department of Electrical and Computer Engineering, Aristotle University of Thessaloniki, Thessaloniki, Greece; ^2^Third Department of Neurology, Medical School, Aristotle University of Thessaloniki, Thessaloniki, Greece; ^3^Department of Electrical and Computer Engineering, Khalifa University of Science and Technology, Abu Dhabi, United Arab Emirates

**Keywords:** Alzheimer's disease, natural language processing, keystroke dynamics, fine motor impairment, machine learning, remote screening, deep learning, smartphone

## Abstract

Mild cognitive impairment (MCI), an identified prodromal stage of Alzheimer's Disease (AD), often evades detection in the early stages of the condition, when existing diagnostic methods are employed in the clinical setting. From an alternative perspective, smartphone interaction behavioral data, unobtrusively acquired in a non-clinical setting, can assist the screening and monitoring of MCI and its symptoms' progression. In this vein, the diagnostic ability of digital biomarkers, drawn from Fine Motor Impairment (FMI)- and Spontaneous Written Speech (SWS)-related data analysis, are examined here. In particular, keystroke dynamics derived from touchscreen typing activities, using Convolutional Neural Networks, along with linguistic features of SWS through Natural Language Processing (NLP), were used to distinguish amongst MCI patients and healthy controls (HC). Analytically, three indices of FMI (rigidity, bradykinesia and alternate finger tapping) and nine NLP features, related with lexical richness, grammatical, syntactical complexity, and word deficits, formed the feature space. The proposed approach was tested on two demographically matched groups of 11 MCI patients and 12 HC, having undergone the same neuropsychological tests, producing 4,930 typing sessions and 78 short texts, within 6 months, for analysis. A cascaded-classifier scheme was realized under three different feature combinations and validated via a Leave-One-Subject-Out cross-validation scheme. The acquired results have shown: (a) keystroke features with a k-NN classifier achieved an Area Under Curve (AUC) of 0.78 [95% confidence interval (CI):0.68–0.88; specificity/sensitivity (SP/SE): 0.64/0.92], (b) NLP features with a Logistic regression classifier achieved an AUC of 0.76 (95% CI: 0.65–0.85; SP/SE: 0.80/0.71), and (c) an ensemble model with the fusion of keystroke and NLP features resulted in AUC of 0.75 (95% CI:0.63–0.86; SP/SE 0.90/0.60). The current findings indicate the potentiality of new digital biomarkers to capture early stages of cognitive decline, providing a highly specific remote screening tool in-the-wild.

## 1. Introduction

Mild Cognitive Impairment (MCI) (1, 2) affects 20% of people over 65 years old worldwide, causing cognitive decline, beyond normal aging, especially in the areas of memory and executive functions, while increasing the probability for the manifestation of the neurodegenerative Alzheimer's disease (AD) (3). People diagnosed with MCI have to be constantly tested over time to ensure that they have not transitioned from mild to severe dementia (4). Although there is currently no treatment for AD, early diagnosis (5) is essential so that the patients, their family and caretakers are better prepared, by making the necessary financial and legal decisions and testing different lifestyle interventions and medication in the MCI or another prodromal stage, to potentially delay or even prevent AD development (6). Studies have indicated that cognitive, behavioral, sensory and motor changes may precede clinical manifestations of AD by several years (7). Current diagnostic methods include a suite of neuropsychological and physiological tests conducted in a clinical setting, estimating key cognitive functions like memory, comprehension, and coordination, alongside laboratory and brain-imaging tests, estimating nerve degeneration and amyloid protein concentration levels (8). However, these validated AD diagnostic methods usually fail to efficiently distinguish early MCI from the normal cognitive trajectory.

The production and comprehension of speech are correlated with the activation and coordination of diverse sensory and cognitive processes in regions of the cerebral cortex, including semantic storage and retrieval, executive functions and working memory (9). Therefore, multiple aspects of the language content, correlated among others with lexical processing, grammatical, and syntactic complexity and word finding, are degrading sharply and rapidly with the progression of AD or the transition from an asymptomatic phase to MCI, compared to healthy ageing (10). Further alarming signs include word class deficits, with noun rates usually decreasing and verb and pronoun rates increasing, as well as limited lexical richness and vocabulary size (11). Efforts to identify and assess linguistic deficits in AD and MCI have mainly been focused on oral speech and its transcripts, either in the form of conversational speech or in narrations, naming tests and picture description tasks. Bucks et al. (12) using measures dependant on word frequencies of lexical items during spontaneous oral speech, managed to discriminate between healthy controls (HC) and people diagnosed with probable dementia, validating the decline in lexical richness and a lower noun rate in the latter group. In parallel, in the various studies analysing connected speech relevant to picture description tasks, reviewed by (13), verbal fluency, semantic processing, and pragmatic language use can be assessed even in prodromal stages, while automatic speech analysis emphasizing on vocal features in the work of (14) reaches an accuracy over 80%. Recent work of (15), using multimodal language data and cascaded classifiers, reveals that the language features alone reach an AUC of 0.70 and their combination with other feature types greatly enhances the system's performance. Tools, like the computer-based software of (16), collecting both textual and acoustic linguistic features from different tasks, facilitate the research toward such direction. As far as written speech is concerned, a longitudinal study of (17) on the texts of three novelists, who developed dementia, indicates the progressive lexical and syntactic changes associated with AD. However, despite the rich research in the area of oral speech, a lack of research in the field of Spontaneous Written Speech (SWS) interactions is evident.

Apart from the speech-related area, there are several indications that cognitive decline in MCI patients is associated, to a certain degree, with motor dysfunction in both lower (18) and upper (19) extremity level functions. Dual-task gait tests, that involve walking while doing a cognitively demanding task, have revealed poor gait performance for amnesic MCI patients (20), while (21) managed to discriminate MCI and HC, with AUC 0.83 and sensitivity/specificity 0.82/0.72, using dual-tasks in a clinical setting and involving a sensor-based upper extremity function motor task instead of walking. The search for useful MCI markers and especially digital biomarkers (22), taking advantage of the mobile and wearable consumer device-derived data and their passive collection (23), is a promising new research field, along the increasing plurality and sensitivity of smart sensors for unobtrusive data acquisition. In this vein, focusing on Fine Motor Control (FMC), variability in typing and finger tapping speed in smartphone screens and computer mouses have been used for early dementia detection, as they present a sharper decline compared to their trajectory in healthy ageing (24, 25). Computer-use behaviors are significantly associated with performance on cognitive and functional assessments, with the temporal characteristics of typing, number of pauses and inter-keystroke intervals having been tested as potential markers for cognitive decline (26). Keystroke dynamics, while typing in computer keyboards and smartphone screens, can be captured in a non-clinical setting and have thus been used for the early detection of multiple conditions, such as Parkinson's disease (27), depression (28), and AD with guided copy tasks (29), noticing again the absence of results in spontaneous unprompted keyboard interactions, that better reflect the natural state of the patient.

Having reviewed separately studies regarding linguistic characteristics and keystroke dynamics, the potential diagnostic properties of the cognitive load, associated and partly overlapping with both written speech production and motor dysfunction remain to be examined. Vizer and Sears (30) use a statistical model of keystroke and linguistic features, extracted from ordinary text-typing activities on a computer keyboard, to monitor signs of early cognitive decline in a PreMCI stage, showcasing the efficacy of a combined feature set, reaching an AUC of 0.80, but using as keystroke features time per key, pause rates and duration and, therefore, not correlating with motor dysfunction symptoms. These findings show the potential of multimodal features and equivalent approaches are needed in an MCI stage, reflecting the dysfunctions in multiple brain regions of the pre-frontal cortex, existent in the early stages of dementia. An overview of selected studies that attempted to detect MCI or AD based on speech and motor deficiency-related data or combinations of the above and their results can be found in Table 1.

**Table 1:** References, feature sets, and results of studies regarding MCI and AD detection based on speech or motor deficiency-related features and multimodal data.

***Feature Set***	***Results***
**(** **12** **)**	
Noun- (N), pronoun- (P), adjective- (A), verb- (V) rate, type token ratio (TTR), Brunet's index (W), Honore's statistic (R), clause-like semantic unit rate (CSU) in transcribed conversational speech	87.5% correct classification, between individuals with probable dementia of Alzheimer type (DAT) and healthy controls, DAT participants had higher mean P, A, V- rate, lower N-rate, higher mean W, lower mean R and TTR, mean CSU did not differ
**(** **14** **)**	
Mean, median, ratio mean, standard deviation of voice and silence duration, periodic vs. aperiodic speech, vocal reaction time, amount of insertions-deletions, irregularity, semantic verbal fluency during short recordings of vocal tasks in-the-clinic	79% accuracy with 20% equal error rate (EER) in classifying MCI vs. HC, 87% accuracy with 13% EER in classifying AD vs. HC with equal SP-SE
**(** **17** **)**	
TTR, lexical repetition, N and V specificity, word class deficit, fillers, syntactic complexity with mean length of utterance, mean number of clauses per utterance, parse tree depth, Yngve depths, D-level scale, use of passive voice in fully parsed texts of three British novelists healthy or with AD	Vocabulary size, repetition and specificity measures validated pronounced decline for authors with AD, syntactic and passive voice analysis did not yield linear results
**(** **13** **)**	
Semantic content, information conciseness efficiency, lexical diversity, total number of words, syntax, V over N rates, coherence prosody, fluency, speech rate in connected speech studies with picture description tasks	Semantic content and conciseness of information yield the best results in detecting MCI and mild AD with picture description tasks
**(** **24** **)**	
Touch and off phase during a finger tapping task of 15 s	SP-SE 0.91–0.52 for ruling out cognitive impairment
**(** **26** **)**	
Mouse operations (amount and time of clicks), keystrokes (amount and timing of text and operational keystrokes), total duration of computer use and pauses during semi-directed computer tasks within a 2-h single testing session	AUC 0.8–0.92 for different features and task combinations with SP 0.62–0.91 and SE 0.8–0.95
**(** **21** **)**	
Motor function speed and variability as measured with two gyroscopes attached to the wrist and upper-arm of the dominant hand during dual tasks (move hand-count numbers) in-the-clinic	AUC 0.83 with SP-SE 0.72-0.82 in predicting MCI and AD
**(** **15** **)**	
Language (26), speech (12), eye movement (22), comprehension (11) features from audio recordings, text transcripts, comprehension questions, and eye tracking during reading silently, aloud and picture description tasks in-the clinic	Combined features: AUC 0.71 and SP-SE 0.79–0.55, Picture description task: AUC 0.72 and SP-SE 0.67–0.63, Verbal reading task: AUC 0.79–0.82 and SP-SE 0.72–0.65, Silent reading task: AUC 0.88 with SP-SE 0.85–0.78 in task fusion
**(** **31** **)**	
370 linguistic (syntactic complexity, grammatical constituents, vocabulary richness, repetitions, information content) and acoustic features (MFCCs) from short narrative samples of the DementiaBank	Top 35 features: 81% accuracy in distinguishing people with AD from HC All the features: 58% accuracy
**(** **30** **)**	
Keystroke (timing, pauses, rates of words, sentence lengths) and linguistic (unique words rate, word class rates, words indicating emotions and cognitive complexity) features from computer-typed texts from older adults with and without PreMCI	Linguistic features: 60% accuracy Keystroke timing features: 68.6% accuracy Combined features: 77.1% accuracy with SP-SE 0.83–0.7 and AUC 0.8

*MCI, Mild Cognitive Impairment; AD, Alzheimer's Disease; HC, Healthy Controls; AUC, Area Under the Curve; SP-SE, Specificity–Sensitivity*.

Motivated by the aforementioned, the aim of this study is to provide an automated method that can identify MCI individuals and distinguish them from HC, based on digital biomarkers, through their routine interaction with a smartphone keyboard in a non-clinical setting. Specifically, it is firstly assessed how cognitive decline is linked with the natural language processing (NLP) of linguistic features of spontaneous written speech (SWS) production and sequentially with the keystroke information regarding specific motor impairment symptoms. Then, their combination is examined with ensemble models, aiming to capture the interlinked dysfunctions in the respective brain regions associated with language and motor skills; thus, improving the overall classification performance. The promising results, when the proposed approach was tested on data from HC and MCI patients, show that such an analysis could provide with predictive analytics of early stages of cognitive impairment, taking into consideration the pragmatic conditions of everyday living. This will contribute in automatic, unobtrusive, remote monitoring, and recommendation of MCI and AD diagnosis.

## 2. Materials and Methods

### 2.1. Data Collection

#### 2.1.1. Participants

The participants of this 6-month long study were recruited in the Day Center “Saint John” of the Greek Alzheimer Association and comprised two groups consisting of in total 12 HC, with the official diagnosis of Subjective Cognitive Impairment (SCI) related to the effects of normal aging and a group of 11 patients diagnosed with MCI. During the duration of the study, there was no clinical progression of the participants' condition. All participants had undergone the same clinical assessment for the official diagnosis within the last 3 years, that included as measures the Mini Mental State Examination-MMSE (32), the Functional Rating Scale for Symptoms of Dementia-FRSSD (33), and the Functional Cognitive Assessment Scale-FUCAS (34), all translated in the participants' native language and scored by medical professionals. The participants were also evaluated in the two main scales related to anxiety and depression, the Beck Depression Inventory-BDI (35) and the Geriatric Depression Scale-GDS (36), with 50% of MCI patients having being identified with minimal to mild depression, while the HC scored negatively. Lastly, blood and neuroimaging tests were conducted and all the study subjects were found negative to Parkinson's Disease. All the participants were 60–75 years old, had finished secondary or higher education and had all been using a smartphone for more than 12 months prior to the study. The groups did not differ in age, gender, and education levels, based on the two-sided Mann–Whitney *U*-test for the age and Chi-squared test for the gender and the education levels (*p* > 0.05). The demographic information of all participants is also presented in Table 2.

**Table 2:** Demographics of participants (MCI, Mild Cognitive Impairment; HC, Healthy Controls) including number of participants, gender (F, female; M, male), age and education level (2: secondary education, 3: higher education, 4: masters-PhD).

**Demographics**	**MCI**	**HC**	**Statistical significance (*p*-value)**
*n*	11	12	N.A
Gender F:M (%)	9:2 (81.8:18.2%)	7:5 (58.3:41.6%)	n.s (*p*)
Avg. Age (std)	67.2 (5.96)	66.2 (4.72)	n.s.(*p* = 0.41)
Avg. Education level (std)	2.63 (0.67)	2.75 (0.62)	n.s.(*p*)

*N.A., not applicable; n.s., not significant with p > 0.05*.

#### 2.1.2. Study Protocol

Participants downloaded in their own smartphones the “Type of Mood” mobile application from Google Play store (37) and created an account, providing information about their gender, date of birth, education, and income levels, smartphone usage, as well as filling in the digitized version of the PHQ-9 questionnaire on depression (38), while giving their consent in their data processing within the app. The study was reviewed and approved by Greece, Bioethics Committee of the Aristotle University of Thessaloniki, Medical School, Thessaloniki, Greece (359/3.4.17) and all participants provided their written informed consent to participate in this study. Sequentially, they activated the app's custom keyboard, which replaced the default typing input method across all aspects of the Android Operating System. The keyboard recorded keystroke timing information, i.e., sequences of timestamps of key presses and releases, as well as typing metadata (delete rate, pauses, number of characters typed, and typing sessions' duration), in the background, without interfering with participants' routine typing and without capturing the characters typed, rendering the process privacy-aware. For each typing session (keyboard shown and afterwards hidden, with at least one key tap in the meantime), the above-mentioned keystroke timing information was stored in a JSON format and indexed as database entries in a local SQLite database, available only to the application. The application would periodically transmit database entries to a remote cloud server (Microsoft Azure), when the user's device was connected to Wi-Fi and charging, accompanied by the uniquely coded ID of the user. Within 6 months, 4,930 typing sessions were collected, 3,000 from 11 MCI patients and 1,930 from 12 HC (more than 100 sessions per individual smartphone), while 3,139 of them, with more than 40 key presses per session, were eventually used for the analysis.

Simultaneously, participants were asked to type down on their phones up to 4 short texts, around a paragraph in length, to be used for the NLP analysis. To simulate SWS production the suggested topics included: (1) a message to a loved one or a good friend, (2) a description of one's day, (3) giving advice on someone, and (4) narrating a short story from a happy memory, as those were the things they usually text their social circle about. The participants typed the texts on their phones at home, based on their own availability and without a time limit, to simulate a non-clinical setting. They could not use the auto-correction feature of their phones and they sent afterwards the texts via email. In total, 10 MCI patients contributed 40 texts and 7 HC contributed 28 texts. From all the participants, 10 MCI patients and 5 HC, respectively contributed for the fused features analysis both texts and keystroke information through their typing activities with the custom keyboard.

### 2.2. Models and Experiments

#### 2.2.1. Pre-processing

The texts were delivered by the participants as emails and saved as txt files with the equivalent user ID. The spelling mistakes and accidental punctuation marks were corrected, to avoid interference with the Part-Of-Speech (POS) Tagging process (words receive tags based on their word class type i.e., noun, verb etc.), given they do not represent studied measures in the following experiments. Using a POS-Tagger trained for the Greek language (39), each text was also stored with a CONNL-U dependency parse tree format (40). Dependency parsing is based on the notion that linguistic units, e.g. words, are interconnected with directional links within a sentence. Therefore, the parsing tree reflects the syntactical relation of the words within the sentence, with the “children” words being dependent from the “root” word, while its depth corresponds to the overall syntactical complexity of the sentence.

The timestamped sequences of the typing data, contained in the stored JSON files, were used to extract keystroke dynamics variables, namely the “hold time” (HT-time interval between pressing and releasing a key) and “flight time” (FT-time interval between releasing a key and pressing the next one), as shown:


(1) $$\begin{array}{l} HT_{n}=t_{n}^{r}-t_{n}^{p}\text{ with }n=1,\;2,\;\ldots ,\;N, \end{array}$$

(2) $$\begin{array}{l} FT_{n}=t_{n+1}^{p}-t_{n}^{r}\text{ with }n=1,\;2,\;\ldots ,\;N-1, \end{array}$$

where $t_{n}^{p}$ and $t_{n}^{r}$ refer to the pressing and releasing times, respectively. With appropriate filtering, by keeping the HT values smaller than 700 μs and the FT values smaller than 3 s, the long pauses were excluded and the keystroke timing information was less susceptible to noise. Zero-padding was used to achieve common dimensionality of 100 values in HT and FT arrays for each typing session.

#### 2.2.2. Feature Extraction

##### 2.2.2.1. NLP features

For each text, a set of nine features was extracted, using the Natural Language Toolkit suite of tools and libraries (41), along custom made functions and the mean value of each feature across the different texts of each user was used in the final features array. Specifically, each text was tokenized by breaking it up into words and punctuation marks and the set of unique words, consisting its vocabulary, was further extracted. The lexical diversity and richness of the texts are expressed with three features. The ratio of unique words over the total number of words is calculated as shown below:


(3) $$\begin{array}{l} dvrst=V/W, \end{array}$$

where *W* denotes the total number of words and *V* the total number of unique words, namely the vocabulary. The Brunet Index—“BI” indicates richer language with lower values, independently from the text's length (42) and is calculated as shown below:


(4) $$\begin{array}{l} BI=W^{V^{-0.165}}. \end{array}$$

Honore's Statistic—“HS,” indicating richer language with higher values (43) is calculated as seen below:


(5) $$\begin{array}{l} HS=\frac{100*logW}{1-\frac{hap}{V}}, \end{array}$$

where *hap* denotes the number of hapaxes legomena (number of words appearing only once within a text). The average number of words per sentence is calculated as seen below:


(6) $$\begin{array}{l} wrd\text{\_}sent=\text{sum(w\_sents)/len(w\_sents)}, \end{array}$$

where *w*_*sents* denotes a list with the number of words per sentence as elements. The ratio of the meaningful vocabulary (words that are not part of the Greek stopwords from the NTLK corpus) is expressed as shown below:


(7) $$\begin{array}{l} nonstop=\text{len(content)}/V, \end{array}$$

where *content* refers to the vocabulary words that are not considered stopwords based on the NLTK corpus. Features related to word class deficits were extracted from the POS-Tagged texts. The nouns over verbs ratio is calculated as shown below:


(8) $$\begin{array}{l} NnVrb=nn/vrb, \end{array}$$

where *nn* denotes the number of nouns and *vrb* the number of verbs within the text. The noun ratio and word finding difficulties are expressed as seen below:


(9) $$\begin{array}{l} Nn=nn/\left(vrb+nn\right). \end{array}$$

**Figure 1:**
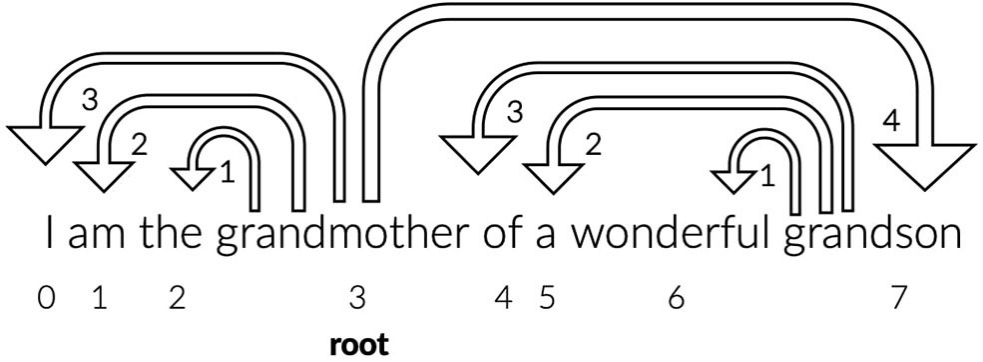
The dependency parsing of the sentence “I am the grandmother of a wonderful grandson” is depicted. The ids of the words, representing the position of the words in the sentence, are indicated with numbers 0–7 and the word “grandmother” is identified by the parser as the root word. The arrows have the direction from the head/root word to the dependent/children, the dependency distance between the pair is indicated on the arrow head and equals the difference of their id values. The words “the,” “am,” “I,” and “grandson” are dependent from the word “grandmother” and the words “wonderful,” “a,” and “of” are dependent from the word “grandson.” The MDD of this sentence based on the Equation (11) equals (3+2+1+4+3+2+1)/7= 2.28.

The pronoun ratio, which quantifies indirect referencing, is presented below:


(10) $$\begin{array}{l} Prn=prn/\left(nn+prn\right), \end{array}$$

where *prn* denotes the number of pronouns within the text. Lastly, a form of the Mean Dependency Distance—“MDD” was used (44) to express the syntactical complexity. Specifically, for each text the MDD of each sentence was calculated separately and then the sum of those MDDs was divided by the number of sentences, getting a mean estimation of this feature for each text. The MDD of each sentence is defined as the sum of the distances of each “child”/dependent word from its “root”/head word over the number of tokens of the sentence as shown below:


(11) $$\begin{array}{l} \begin{array}{c} MDD_{\text{(sentence)}}=\frac{1}{N-1}\overset{N}{\underset{i=1}{\sum }}|DD_{i}|, \end{array} \end{array}$$

where *N* denotes the number of tokens and *DD*_*i*_ the dependency distance from the *i* − *th* link. The latter was calculated with Pre-order Tree Traversal (traverse the root, the left sub-tree, and then the right sub-tree) of the dependency parsed trees format of the sentences in the CONLL-U files. In Figure 1, there is an example of a sentence with dependency parsing.

##### 2.2.2.2. Keystroke features

**Figure 2:**
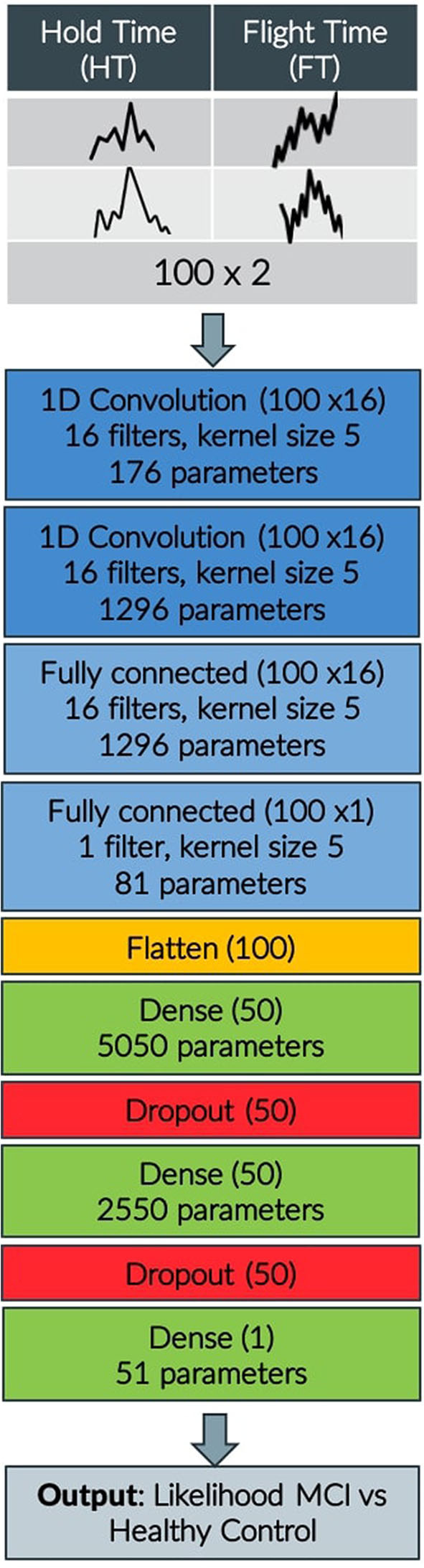
CNN model layout indicating input, convolution, flattening, dense, and dropout layers, output shape of each layer and number of parameters.

As far as the keystroke dynamics are concerned, the extracted features are associated with three indices Bradykinesia (B), Rigidity (R), and Alternate Finger Tapping (AFT), the symptoms describing Fine Motor Impairment (FMI). Specifically, one dimensional CNN-based autoencoders, consisting of two sequentially connected convolutional layers (kernel size of 5, 16 filters) without max-pooling layers, took as inputs the HT and FT sequences of the typing data and were used to learn a neural network's encoding mechanism for representing efficiently the keystroke dynamics and reject the noise. The CNN was trained in an unsupervised manner (80–20% train-test split on 34,000 typing sessions, back-propagation for 50 epochs with mini-batches of size 64, RMSprop optimizer with learning rate of 10^−3^ for the mean squared error loss function) on a dataset of a relevant study of Parkinsonian screening (45), to learn the inherent structure of keystroke dynamics and represent them with a limited number of features. Indicatively, the in-the-wild development dataset, used for unsupervised pre-training of the neural network parameters consisted of 34,000 typing sessions with keystroke dynamics drawn from subjects with age ≤ 40 years (self-reported). A two-layer fully-connected network with 50 hidden nodes was added to the already trained networks and the final network was fine-tuned on an in-the-clinic dataset of 33 subjects, by optimizing regression models with leave-one-subject-out (LOSO) cross validation (50 epochs with mini-batches of size 32) to each estimate the severity of the symptoms Rigidity/Alternate Finger/Bradykinesia in relation with the ground-truth Unified Parkinson's Disease Rating Scale (UPDRS) Part III single-item scores 22/23/31 (46). The number of parameters reaches 10,500 altogether and the overall model layout can be found in Figure 2.

The in-the-clinic development dataset consists of 274 typing sessions in total (up to 10 text-excerpts for each user) with keystroke dynamics from 33 demographically matched subjects [18 early Parkinson Diseases (PD) patients and 15 HC], whom underwent clinical examination by neurologists and their FMI was evaluated by their UPDRS Part III single-item scores 22/23/31, expressing rigidity of upper extremity/alternate finger tapping/general body bradykinesia-hypokinesia, respectively. Before being tested to our dataset, consisting of more than 3,000 typing sessions of MCI patients and HC, the models have also been tested in 4 in-the-wild datasets: (1) a dataset with 216,000 typing sessions with keystroke dynamics from clinically examined 214 subjects (PD vs. HC), with self-reported demographics, through the iPROGNOSIS app, (2) a dataset with 36,000 typing sessions with keystroke dynamics from 39 subjects (PD/HC:22/17), (3) a subset of the previous dataset with 7,600 typing sessions, drawn from *de novo* PD patients and the same HC (*de novo* PD/HC: 9/17), (4) the union of the first two datasets with 252,000 typing sessions with keystroke dynamics from 253 subjects (PD/HC: 67/186) The optimized scores produced indicators that can be used in-the-wild prediction of UPDRS scores 22/23/31, yielding correlation 0.66/0.73/0.58, respectively, in the validation set of 36,000 typing sessions. The trained models were used to infer based on the typing data from 11 MCI patients and 12 HC, and estimations for each symptom (R/B/AFT 0-4) were extracted for each typing session of each user (47). All the features from the natural language and typing processing can be found in Table 3.

**Table 3:** Features from the natural language and typing processing.

**Features**	**Descriptions**
**NLP features**	
dvrst	Lexical diversity
nonstop	Meaningful content
wrd_sent	Avg. No. words per sentence
BI	Brunet index
HS	Honore's Statistic
NnVrb	Noun per Verbs ratio
Nn	Noun ratio
Prn	Pronouns ratio
MDD	Syntactical complexity
**Keystroke features**	
R indices	Rigidity
B indices	Bradykinesia
AFT indices	Alternate finger tapping

#### 2.2.3. Experiments and Classification Models

**Figure 3:**
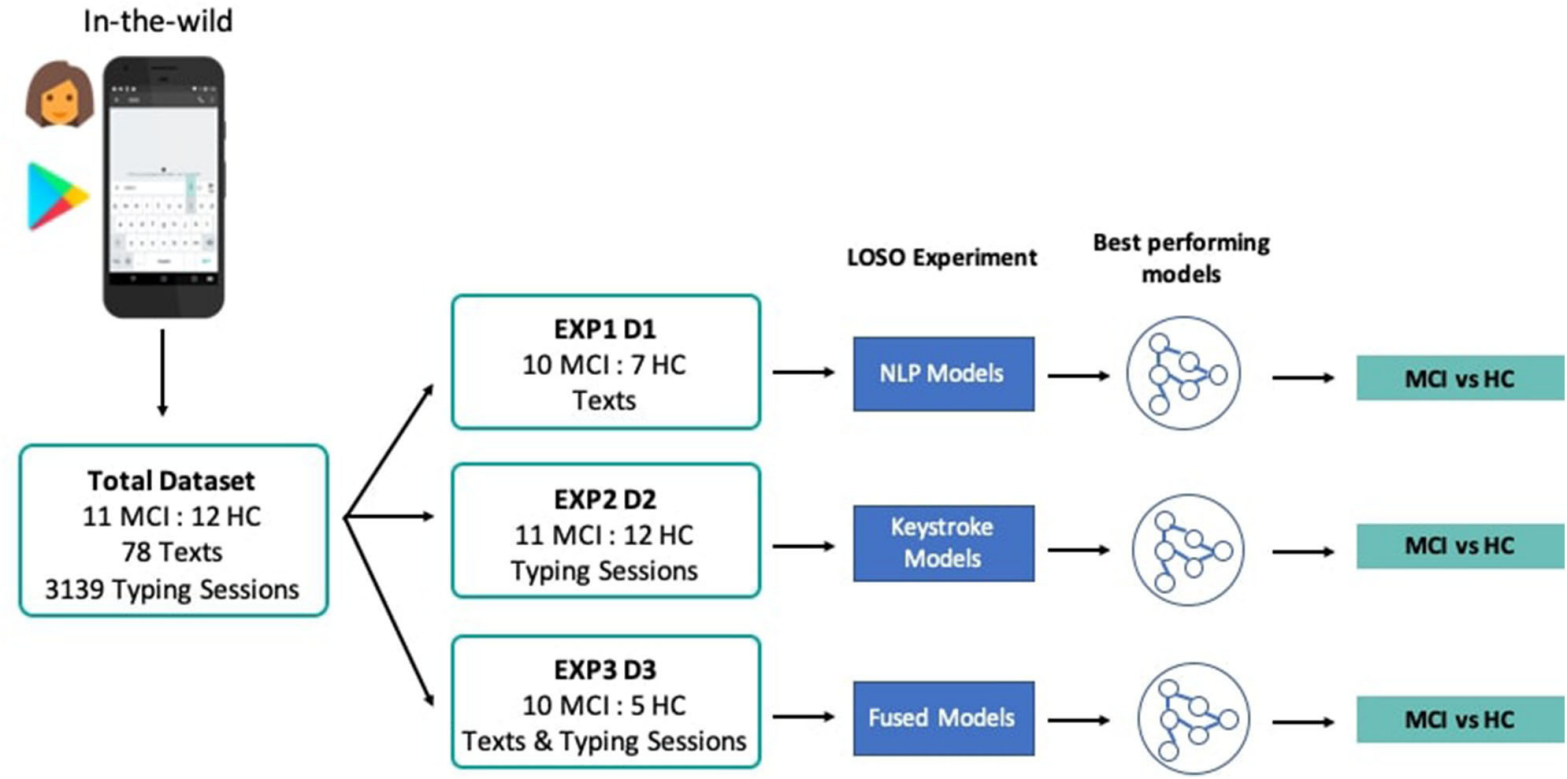
System pipeline and experiments' overview. Texts and typing sessions were collected from MCI patients and HC through the custom keyboard of the TypeOfMood application in a non-clinical setting. The total dataset was split accordingly (D1, D2, D3) to the needs of the three experiments that tested NLP models (EXP1), keystroke models (EXP2), and models with fused feature sets (EXP3). Multiple rounds of LOSO experiments yielded the best performing models to distinguish among MCI and HC.

In order to address the study research questions, that is, the exploration of the classification performance between MCI and HC, using features drawn from the NLP, keystroke dynamics, and combined feature spaces, three experiments, i.e., EXP1, EXP2, and EXP3, with different feature sets and demographically matched sub-cohorts were conducted, accordingly. In each experiment, three different classifiers, Logistic Regression, Random Forest, and k-Nearest Neighbors are evaluated based on their accuracy and the receiver operating characteristic (ROC) analysis, after multiple rounds of LOSO cross-validation. ROC analysis is an iterative process of varying the discrimination threshold of a binary classifier and outputting the (Sensitivity, Specificity) pair for each threshold. The ROC curve is then formed by plotting the output pairs of (1—Specificity, Sensitivity). This analysis provides reliable insights into the performance of a classification model even when datasets are not completely balanced. To assess the statistical significance of classification results, sampling with replacement (1,000 bootstraps) is further used here to define a ROC curve distribution, by obtaining the average value (solid line in figures) and the confidence intervals (shadowed areas in the figures) of the area under the ROC curve (AUC). Where reported, specificity/sensitivity values correspond to the optimal ROC-based cut-off point (decision threshold), estimated by maximizing the Youden Index (48), for equal cost of misclassifying MCI patients and HC. A Univariate Feature Selection process was used, where each feature was individually assessed with regards to its statistical significance with the label and combinations of the features with the highest correlation were chosen for the optimal feature set in each experiment. Standardization and scaling was performed in the feature sets when needed, by removing the mean and scaling to unit variance. An overview of the pipeline and the different experiments and cohorts can be seen in Figure 3.

In particular, at EXP1, 78 texts from 10 MCI patients and 7 HC were used to extract NLP features and test the predictive potential of SWS production. In EXP2, 3,139 typing sessions from 11 MCI patients and 12 HC were used to extract the keystroke related features that quantify FMI symptoms and test their predictive potential linked to cognitive decline. In EXP3, NLP and keystroke feature sets, combined differently in 3 models (A, B, C), were used to assess the performance of fused feature sets against models with separate NLP and keystroke features (“Just NLP,” “Just Keys”), from 10 MCI patients and 5 HC contributing both texts and typing sessions. Model “A” concatenates the probabilities/predictions of the “Just NLP” model with keystroke features and feeds them into another Random Forest classifier, while model “B” concatenates the probabilities/predictions of the “Just Keys” model with NLP features and feeds them into another k-Nearest Neighbors classifier. Lastly, model “C” concatenates the probabilities/predictions of both the “Just Keys” and “Just NLP” models and feeds them into another Random Forest classifier. The cohorts and the chosen optimal feature sets of each experiment can be found in Table 4.

**Table 4:** Cohorts (number of MCI:HC), optimal feature sets and models of the experiments EXP1, EXP2, EXP3.

**Cohort (MCI:HC)**	**Feature set**	**Model**
**EXP1: NLP features**		
10:7	nonstop, dvrst, HS, MDD	N.A
**EXP2: Keystroke features**		
11:12	B and R indices	N.A
**EXP3: Fused features**		
10:5	Nonstop, dvrst, MDD	Just NLP
	B and R indices	Just Keys
	Probabilities of Just NLP & B indices	A
	Probabilities of Just Keys & dvrst, nonstop, MDD	B
	Probabilities of Just NLP & probabilities of Just Keys	C

*N.A., not applicable*.

## 3. Results

**Figure 4:**
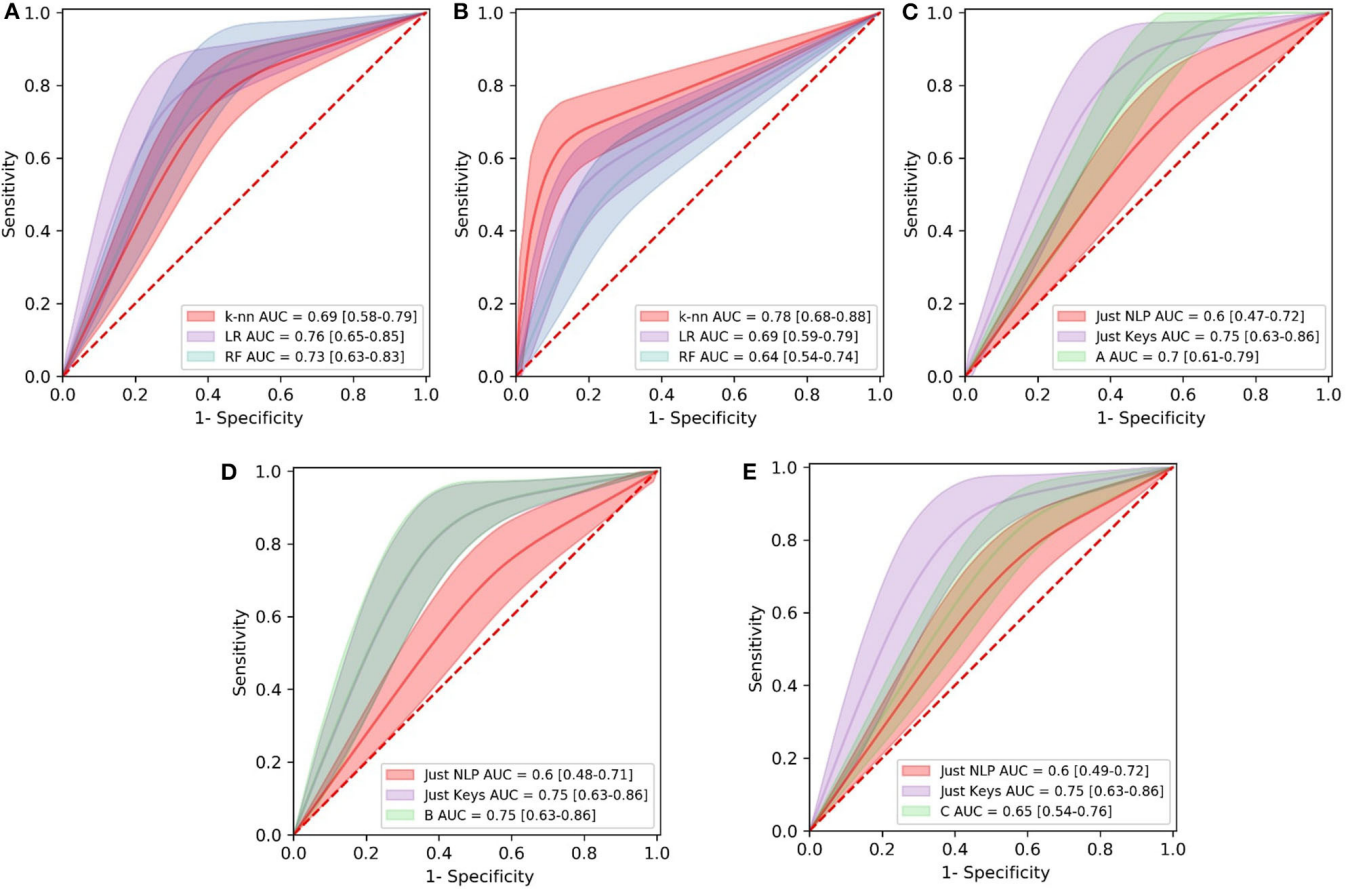
Comparison of Receiver Operating Characteristics (ROC) curves of all the models in the first **(A)**, second **(B)**, and third **(C–E)** experiment. Area Under the Curve (AUC) values for models are shown with 95% Confidence Intervals. LR, Logistic Regression; RF, Random Forest; knn, k-Nearest Neighbors.

Table 5 along with Figure 4 summarize the acquired results from all three experiments. In the following subsections, the specific results per experiment are presented.

**Table 5:** Results of the three experiments with NLP features, keystroke features and their combination in cascaded classifiers.

**Classifier**	**Accuracy**	**AUC**	**Specificity**	**Sensitivity**	**Model**
**EXP1**					
**LR**	**0.76**	**0.76**	**0.80**	**0.71**	**N.A**
RF	0.71	0.73	0.83	0.43	N.A
k-nn	0.71	0.69	0.80	0.57	N.A
**EXP2**					
LR	0.70	0.69	0.54	0.83	N.A
RF	0.66	0.65	0.55	0.75	N.A
**k-nn**	**0.77**	**0.78**	**0.64**	**0.92**	**N.A**
**EXP3**					
LR	0.67	0.60	0.80	0.40	Just NLP
k-nn	0.80	0.75	0.90	0.60	Just Keys
RF	0.75	0.68	0.90	0.40	A
**k-nn**	**0.80**	**0.75**	**0.90**	**0.60**	**B**
RF	0.73	0.65	0.90	0.40	C

*Bold values denote the highest performing models. N.A., not applicable*.

### 3.1. First Experiment

Combinations of the features “nonstop,” “dvrst,” “HS,” and “MDD,” comprising the optimal feature set, were used with the different classifiers. The Logistic Regression classifier has an AUC of 0.76, accuracy 76% and specificity/sensitivity of 0.80/0.71, respectively. The Random Forest Classifier has an AUC of 0.73, accuracy 71% and specificity/sensitivity of 0.83/0.43 respectively. The k-nn classifier with seven nearest neighbors has an AUC of 0.69, accuracy 71% and specificity/sensitivity of 0.80/0.57, respectively. The Logistic Regression classifier appears to have the best performance, whereas the Random Forest one, due to the small number of data wasn't able to perform that well. We observe higher specificity than sensitivity levels. The three ROC curve distributions of the first experiment can be found in Figure 4A.

### 3.2. Second Experiment

Combinations of the “B” and “R” indices, comprising the optimal feature set, were used with the different classifiers. The Logistic Regression classifier has an AUC of 0.69, accuracy 70% and specificity/sensitivity of 0.54/0.83, respectively. The Random Forest Classifier has an AUC of 0.65, accuracy 66% and specificity/sensitivity of 0.55/0.75, respectively. The k-Nearest Neighbors classifier with seven nearest neighbors has an AUC of 0.78, accuracy 77% and specificity/sensitivity of 0.64/0.92, respectively. The k-Nearest Neighbors classifier appears to have the best performance and we observe higher sensitivity than specificity levels. The three ROC curve distributions of the second experiment can be found in Figure 4B.

### 3.3. Third Experiment

The “Just NLP” model resulted in an AUC of 0.60, accuracy 67% and specificity/sensitivity of 0.80/0.40, respectively. The “Just Keys” model resulted in an AUC of 0.75, accuracy 80% and specificity/sensitivity of 0.90/0.60, respectively. Model “A,” resulted in an AUC of 0.68, accuracy 75% and specificity/sensitivity of 0.90/0.40, respectively (Figure 4C). Model “B” resulted in an AUC of 0.75, accuracy 80% and specificity/sensitivity of 0.90/0.60, respectively (Figure 4D). Model “C” resulted in an AUC of 0.65, accuracy 73% and specificity/sensitivity of 0.90/0.40, respectively (Figure 4E). It is validated that the feature sets that have combined the potential of both NLP and keystroke dynamics have better performance, even though the dataset tested and the number of data subjects was quite limited and we observe, once more, higher specificity than sensitivity levels. The highest performance in the current dataset is accredited to model “B,” with similar results with the “Just Keys” model, while model “A” also has very high specificity levels.

## 4. Discussion

Digital and Connected Health is an emerging field, encompassing novel, efficient, effective, and accessible technological tools, that could contribute greatly to early disease diagnosis and management. The aim of this study has been the development of an objective tool for the detection of MCI patients against HC, by exploiting linguistic characteristics and keystroke dynamics, during routine typing on mobile touchscreens. The adopted design reflects the natural spontaneous state of the users and its unobtrusive data collection fashion meets the need of long-adherence. Simultaneously, such a tool assists the longitudinal passive monitoring and diagnosis of the condition, in an interpretable way, facilitating physicians and medical interpretation, through high-frequency sampled data streams. Overall, the study further verifies the relationship between cognitive functions and motor dexterity, being consistent with the overlapping cognitive and motor neural circuitry in the brain, and paving the way toward new approaches for dementia risk screening.

A number of studies have shown that speech and language, being ubiquitous in everyday communication, can provide early signs of MCI and other prodromal stages of AD (49), while being correlated with a lapse in episodic and semantic memory. Episodic memory refers to the multifaceted process that enables the retrieval of detailed evocative memories from the past, while semantic memory is linked with the retrieval of general conceptual knowledge divested of specific spatiotemporal contexts (9). Word finding difficulties and pronounced word class deficits are considered as some of the earliest manifestations of language breakdown in MCI and AD and implicate loss of semantic knowledge and difficulties in encoding new information (12). Our study further validates these impairment patterns per MCI subject, with lower mean number of nouns [both overall (*Nn*) and when compared to verbs (*NnVrb*)], and higher mean number of pronouns (*Prn*) in MCI patients compared to HC, despite the fluctuation noticed in the number of words per sentence within subjects over successive text sessions (*wrd*_*sent*). The measures of the vocabulary size and lexical diversity (*dvrst*, *BI*, *HS*) were decreased within the group of MCI patients as expected (11), with slightly higher mean values of the *BI* and greatly lower values of the *HS*, indicating pronounced lexical repetitions with less hapaxes and lexical richness deficits for the MCI patients. Regarding the *nonstop* metric, that was part of all optimal feature sets in all experiments, the MCI patients appeared to use less connecting words/stopwords, reflecting potentially a decreased syntactical complexity, as an expected remedy of their memory loss capacity (50), that was further validated with the lower mean values of the *MDD* metric. Although the *MDD* metric has been primarily used to assess the general complexity of different languages (44), we are using it for the first time on a micro-level and it managed, in our context, to efficiently reflect the syntactical complexity of written speech per subject.

Regarding motor dysfunction in the upper extremities in early stages of dementia, several studies suggest that mild Parkinsonian signs are associated with MCI patients (51) and the degree of motor impairment may help identify those at risk for AD (18). Our machine learning-based estimation of dominant hand bradykinesia (slowness of movement) and rigidity (muscle stiffness) indices managed to efficiently distinguish between MCI patients and HC, validating the connection between FMI and cognitive decline, given that none of our participants was diagnosed with PD. The diagnostic potential of keystroke dynamics has been proven in previous studies (52) and our results of longer, more variant pressing of the keyboard keys and slower finger coordination across the screen for the MCI patients, further strengthens this potential. These findings are also aligned with studies experimenting with finger tapping speed (24), associated with short-memory lapses and other fine motor dexterity (53) and upper extremity function (21) tests conducted in a clinical setting (data in-the-clinic). Nevertheless, our approach goes several steps further by encapsulating the natural state of the users, capturing non-invasively and longitudinally their typing sessions, without the need for special technical equipment and during their pragmatic real-life activities (data in-the-wild), thus enabling a continuous monitoring of the very early stages of the condition.

Taking it a step further, the study evaluated the combined diagnostic potential of the NLP and keystroke related features, in various models, to validate the clinical suggestions that hand dexterity and FMI co-exist with MCI, possibly sharing similar pathogeneses (54). The combined feature sets in EXP3 had indeed an increased performance in detecting MCI patients from HC, especially model *B* that combined cascadedly the predictions of the *JustKeys* model of solely keystroke features with NLP features, being aligned with other studies evaluating multimodal data combinations (15, 30). The rationale of this association of features resides in the fact that hand dexterity requires complex cognitive processes, beyond sensorimotor coordination of the limbs with the eyes, linked with executive functions, such as attention, judgement, planning, and memory (20). Our results further reinforce the relationship between cognitive and motor function in other functional activities, such as written speech production in our context, beyond the typical mobility tasks and self-reporting as suggested by (19), with a novel FMI detection tool. Moreover, SWS production activates primarily the prefrontal cortex of the brain, associated with both executive cognitive functions and hand dexterity, while being consistent with the overlapping and reciprocal neural circuitry of motion and cognition in the cerebellum and the subcortical structures (55). Therefore, our fused models reflect the overall intricacies related to SWS production, as the thought and cognition process, linked with motor deficiencies, materialize to the actual speech production, providing a highly valuable patient phenotype. The high specificity levels of the NLP models are further enhanced with the highly sensitive and granular information coming from the greater amount of typing sessions against the fewer text samples, resulting in a robust system. In a correlation analysis (using the Pearson correlation coefficient) between the clinical scores of MMSE, FUCAS, and FRSSD scales and the predictions of our models and individual features, the bradykinesia indices significantly correlated (*r*= −0.56, *p* < 0.01) with the MMSE clinical scores and the predictions of the NLP models significantly correlated (*r*= −0.55, *p* < 0.01) with the FUCAS clinical scores. These correlations tangibly showcase the connection of the FMI symptoms, like bradykinesia, with the clinically verified cognitive decline, aligned with the relevant literature (19, 51, 53), and showcase how linguistic deficiencies, captured with the NLP analysis, reflect clinically measured functional deficiencies in everyday life tasks (9), as those used for the FUCAS measure. The estimated correlation levels (around 0.5) between the overall predictive models and the chosen measures were anticipated, since these scales have low sensitivity and variation within the MCI spectrum and the diagnosis at such an early stage is greatly dependant from additional neuroimaging and physiological tests beyond these individual scores.

The novelty of the current study is that it sets up an interpretable framework of unobtrusive assessment of individual symptoms of the early stages of dementia and MCI, harvesting data in-the-wild and thus reflecting the natural state of the user, while still reaching for high correlation with the equivalent clinical scores. The latter is of high importance toward personalized monitoring of different risk factors preceding clinical diagnosis, while the detection of the severity level of FMI and cognition-related symptoms could also facilitate personalized interventions for better management of the patient's condition and increased quality of life. In parallel, such analyses are linked with the use of smartphones and virtual keyboards, assisting further the mobile health booming. The high specificity levels of the proposed models are aligned with the requirements of a remote monitoring system that needs to limit “false alarms” for the HC and the CNN architecture paves the way for new methodologies in digital diagnostic systems. Future work could examine whether interventions targeting neuromuscular traits, such as hand and motor dexterity, may also benefit higher cognitive and functional outcomes. Furthermore, the combination of other data sources, e.g., acoustic features of oral speech (31), gait performance (56), behavioral and social metrics (57), along the further sophistication of the linguistic features extraction, can yield greater performance. Moreover, scaling the study to a larger pool of clinically validated subjects, along a continuous data stream acquisition, will lead to even more robust values in diagnostic performance and thus assist physicians in their clinical estimations and treatment responses of the condition. Therefore, beyond the micro-level approach of this tool, targeted to the user's needs, the benefits extend to the realm of precision medicine toward more efficient clinical decision making.

### 4.1. Limitations and Implications

Despite the promising results presented here, there are some limitations to be considered. Firstly, the overall size of the cohort was small as the study demanded specific educational levels, technological familiarization, and an extensive suite of neuropsychological and physiological tests for both MCI patients and HC. Clearly, this resulted in a difficult recruitment process at the specific age group. Nevertheless, the models have been designed with scalability in mind and re-analyzing data collected from a larger cohort will lead to a more accurate and robust performance. The validity of the self-reported demographics of the study's participants could also be considered as another limitation, but these characteristics did not yield any statistical significance and therefore could not greatly affect the end result. As far as the NLP analysis is concerned, large population observational studies with a greater amount of text samples are required to account for inter- and intra- subject variability in written speech production, given the expected heterogeneity in linguistic changes among individuals in both normal ageing and dementia. Although linguistic decline is accelerated in the presence of MCI, these changes are also highly correlated with the educational and literacy level and familiarization with written speech production, demanding a longitudinal monitoring of speech characteristics of the participants for more generalized results. Moreover, syntax and word choice is also dependent on the stylistic choice of each participant's writing style and thus may account for differences in the metrics that are linked with syntactical complexity. Nevertheless, here we focused on metrics that could yield diagnostic potential despite the limited number of texts and reflect the most common linguistic changes linked to cognitive decline and analyzed the data, bearing in mind these constraints. Furthermore, errors of the Greek POS-Tagger in the part of speech tagging process, although rare, may occur and thus can affect the accuracy of the syntactical parsing process and other measures like the noun, pronoun and verb ratios. As far as the keystroke dynamics analysis is concerned, the algorithms identifying early FMI symptoms were trained on an extensive PD and HC cohort of a relevant study (45), as the MCI and HC cohort of this study was relatively small. However, this does not interfere with the validity of the process, as the algorithms detect the motor symptoms' severity and not Parkinson's condition itself, even though clinically these symptoms do have similarities in these two neurodegenerative diseases (51). Moreover, the results have been calculated with a confidence interval to take into account potential deviations and the longitudinal nature of the data, collected in a non-clinical setting during the routine typing of participants, gave us an ecologically valid and more realistic picture of the FMI symptoms. Lastly, although this study can be materialized as a diagnostic tool to be used at a clinical setting, privacy, and security issues related to the written speech content and the medical data of the participants have to be catered properly, while abiding with all the guidelines for digital health tools (58).

## 5. Conclusion

In this work, a new perspective in the detection of MCI, based on natural language and touchscreen typing processing during SWS production, was presented. Linguistic features, keystroke dynamics and their fusion, reflecting both cognitive and motor deficiencies and their reciprocal expression in MCI patients, are assessed as digital biomarkers. Experimental results in demographically matched cohorts and machine learning models have justified an efficient discrimination performance for the fused feature sets (AUC ≥ 0.75), that provide a complete phenotype of MCI-related symptoms. The promising results presented here pave the way toward a holistic, objective patient-centric AD detection tool to be even successfully deployed in a non-clinical setting.

## Data Availability Statement

The datasets presented in this article are not readily available because they cannot be publicly uploaded and accessed without a reasonable request. Requests to access the datasets should be directed to Leontios J. Hadjileontiadis, leontios@auth.gr.

## Ethics Statement

The studies involving human participants were reviewed and approved by the Bioethics Committee of the Aristotle University of Thessaloniki, Medical School, Thessaloniki, Greece (359/3.4.17). The patients/participants provided their written informed consent to participate in this study.

## Author Contributions

AN, DI, and LH conceived the study protocol and contributed to the manuscript. AN, DI, SH, and VC developed the keyboard and the keystroke dynamics processing algorithms. AN developed the NLP feature extraction and processing algorithms and conducted all experiments. MT and Alzheimer Hellas center conducted the clinical evaluations. AN and DI analyzed the data, developed the participants demographic information, participant information and consent process, and handled the data governance procedures as well as the corresponding ethic approvals. All authors discussed the results. All authors contributed to the article and approved the submitted version.

## Conflict of Interest

The authors declare that the research was conducted in the absence of any commercial or financial relationships that could be construed as a potential conflict of interest.
